# Serial prophylactic exchange blood transfusion in pregnant women with sickle cell disease (TAPS-2): statistical and qualitative analysis plan for a randomised controlled feasibility trial

**DOI:** 10.1186/s13063-023-07235-x

**Published:** 2023-03-24

**Authors:** Paul T. Seed, Sarah B. Brien, Laura L. Oakley, Vicky Robinson, Joseph Sharif, Hilary Thompson, Jeannine Joseph, Eugene Oteng-Ntim

**Affiliations:** 1 Division of Women’s Health, King’s College London, 10th floor North Wing, St Thomas’ Hospital, Lambeth Palace Road, London, SE1 7EH UK; 2 School of Primary Care, Population Sciences and Medical Education, Faculty of Medicine, University of Southampton, Southampton General Hospital, Southampton, SO16 6YD UK; 3 Department of Non-Communicable Disease Epidemiology, London School of Hygiene and Tropical Medicine, Keppel Street, London, WC1E 7HT UK; 4 Centre for Fertility and Health, Norwegian Institute of Public Health, Skøyen, P.O. box 222, N-0213 Oslo, Norway; 5 Guy’s and St Thomas’ NHS Foundation Trust, Westminster Bridge Road, London, SE1 7EH UK; 6 Manchester University NHS Foundation Trust, Oxford Road, Manchester, M13 9WL UK; 7 Patient author, c/o Guy’s and St Thomas’ NHS Foundation Trust, Westminster Bridge Road, London, SE1 7EH UK

## Abstract

**Background:**

There are significant knowledge gaps regarding the effectiveness of serial prophylactic exchange blood transfusion (SPEBT) for pregnant women with sickle cell disease (SCD). The protocol for the randomised feasibility trial assessing SPEBT versus usual care in women with SCD (TAPS2 trial) has previously been published. This publication outlines the statistical and qualitative analysis plan for the study.

**Methods and design:**

TAPS2 is a randomised two-arm phase 2 feasibility trial with a nested qualitative study and health economic evaluation*.* Up to 50 pregnant women with SCD and a singleton pregnancy will be recruited and individually randomised to either SPEBT approximately every 6–10 weeks until delivery (intervention arm) or to usual care (control arm). Information will be collected on a range of feasibility and clinical outcomes.

**Results:**

Due to the impact of COVID-19 on study recruitment, the initial study period of 24 months was extended to 48 months. Other protocol updates designed to mitigate the impact of COVID-19-related disruption included allowing for remote consent and conducting all qualitative interviews by telephone. The primary outcome for the trial is the overall recruitment rate. The number of women screened, eligible, consented, randomised and withdrawn will be summarised as a CONSORT flow diagram. Differences in clinical outcomes will additionally be presented as an initial assessment of efficacy and to inform sample size calculations for a future definitive trial. Qualitative interviews with trial participants and clinicians will be analysed using reflexive thematic analysis; data from interviews with participants who declined to participate in the trial will be extracted and incorporated into summary tables to report key findings. The health economic analysis plan is not covered by this update.

**Conclusion:**

The publication of this analysis plan is designed to aid transparency and to reduce the potential for reporting bias.

**Trial registration:**

NIH registry (www.clinicaltrials.gov), registration number NCT03975894 (registered 05/06/19); ISRCTN (www.isrctn.com), registration number ISRCTN52684446 (retrospectively registered 02/08/19).

**Supplementary Information:**

The online version contains supplementary material available at 10.1186/s13063-023-07235-x.

## Background

Pregnancies in women with sickle cell disease (SCD) are associated with a higher risk of sickle and pregnancy complications [1, 2]. There are limited options for treating SCD during pregnancy. Serial prophylactic exchange blood transfusion (SPEBT) has been shown to be effective in treating SCD outside pregnancy [3], but there is a lack of evidence regarding its use during pregnancy. The aim of the TAPS2 study is to assess the feasibility and acceptability of conducting a future phase 3 trial investigating whether SPEBT in pregnant women with SCD improves maternal and foetal outcomes. The study protocol for TAPS2 has previously been published [4]. Results will be reported according to the recommendations of the CONSORT group, as extended to randomised pilot and feasibility studies [5, 6]. In this manuscript, we present the TAPS2 statistical and qualitative analysis plan, covering the feasibility outcomes and qualitative analysis. The health economics analysis is described in a separate document available on request from the authors.

## Summary of study 

TAPS2 is a randomised two-arm phase 2 feasibility trial with a nested qualitative study and health economic evaluation*.* Pregnant women with SCD and a singleton pregnancy, who are at or below 18 weeks gestation, are recruited from either the SCD clinics or antenatal booking clinics at participating maternity units in England. Eligible women who consent to participate in the feasibility trial are individually randomised (ratio 1:1) to the intervention or control arm via the study-specific internet-based secure data management system (MedSciNet™). Participants will be randomised using minimisation, balancing on centre, SCD genotype, and maternal age category. All recruited women will receive the usual NHS antenatal care for pregnant women with SCD, based on the Royal College of Obstetrics and Gynaecology (RCOG) guidelines (published in 2011), and latterly the British Society for Haematology (BSH) guidelines (published in 2021) [7, 8]. Women allocated to the intervention arm will additionally receive SPEBT, starting within 2 weeks of randomisation. SPEBT will be performed using automated erythrocytopheresis, approximately every 6–10 weeks until delivery, with the aim of maintaining HbS% or combined HbS/HbC% below 30%. Women in the control arm will only receive transfusion if clinically indicated.

## Study objectives

### Primary objective

The primary objective is to assess the feasibility of recruitment into a future phase 3 trial by assessing the willingness of pregnant women with SCD to take part in a randomised controlled trial comparing SPEBT to standard care.

### Secondary objectives

The secondary study objectives are as follows:

- Identify barriers and facilitators to participation in the trial, including assessing reasons for refusal, from study participants, clinicians and, where possible, those unwilling to participate.
- Assess retention rates of participants throughout pregnancy in both arms of the study.
- Identify reasons for attrition.
- Assess the willingness of clinicians to recruit into this trial.
- Assess the proportion from the control arm advised clinically to start prophylactic blood transfusion.
- Measure clinical outcomes for women and infants including an initial preliminary assessment of efficacy for a future definitive trial.
- Generate data to inform the design of a definitive trial, including identifying the primary outcome and sample size for the definitive trial.
- Record safety issues around blood transfusions in both arms of the study.
- Identify strategies to optimise recruitment and retention.
- Assess the acceptability of the intervention, trial procedures and conduct, including identifying the core outcomes that are considered important to measure.
- Assess participants’ experience of taking part in the study.
- Explore the cost implications of the proposed intervention and to assess measurement tools and methods.
- Assess two widely used HRQoL measures against each other.

## Sample size

Details of the original study sample size calculation were included in the previously published protocol. Briefly, we estimated that a sample of 40 women (20 in each arm) would allow us to estimate the overall recruitment rate per woman with SCD to within 10% of the true value or better using the Clopper-Pearson exact Binomial method for a 95% confidence interval. Assuming a 50–60% recruitment rate and an estimated 20% loss to follow-up, we planned to recruit 50 participants over an 18-month period in order to reach a final sample of 40 women.

## Trial recruitment and data collection

Pregnant women with SCD who potentially meet the inclusion criteria [4] will be provided with the Patient Information Sheet (PIS) and invited to meet with the site research practitioner. Written informed consent will be obtained by one of the trial physicians or practitioners. Baseline data collection will include current and previous medical and obstetric history and socio-demographic information. Information on maternal, sickle and foetal complications will be collected every 4 to 6 weeks during pregnancy. Health economic data will be collected at least once each trimester and postnatally using the 3L and 5L Euroquol EQ-5D questionnaires [9]. Details of health in late pregnancy, labour and birth, postnatal complications and neonatal status will be extracted from medical records. At 6 weeks postpartum, participants will be asked to provide additional information on postpartum complications and health.

## Protocol updates

The study received NHS ethics approval in March 2019 (London – Surrey Borders Research Ethics Committee, REC reference 18/LO/2070). Following ethical approval, recruitment commenced in five maternity units in England: four in inner London (Guy’s and St Thomas’ NHS Foundation Trust, King’s College Hospital NHS Foundation Trust, St George’s University Hospitals NHS Foundation Trust, Whittington Health NHS Trust), and one in Manchester (Manchester University NHS Foundation Trust). Two further sites were added in 2021 (University Hospitals of Leicester NHS Trust, Leicester; Imperial College Healthcare NHS Trust, London), resulting in a final total of seven participating units.

The study started in April 2019 and the first trial participant was recruited in July 2019. In March 2020, recruitment at all active sites was suspended due to the developing COVID-19 pandemic. All sites remained closed to recruitment for a minimum of 4 months, after which individual sites experienced further closures due to the direct and indirect effects of the COVID-19 pandemic. Our planned 18-month recruiting period was adjusted to allow for these pauses in recruitment.

A number of changes were made to the protocol to mitigate the impact of COVID-19-related disruption to the study. In October 2020, enrolment procedures were adapted to allow for remote consent. Although we had initially planned to conduct some qualitative interviews face-to-face, we switched to conducting all interviews by telephone. A further amendment was approved in August 2021 allowing the creation of a TAPS2 microsite to aid remote recruitment of potential participants.

The TAPS2 study was initially planned to last 24 months. A 12-month costed extension was awarded in May 2021 providing a new study end date of April 2022. An additional non-costed 12-month extension was granted in February 2022 to permit TAPS2 to continue until April 2023.

All the above changes to the protocol were submitted and approved as amendments to the original study protocol.

## Statistical analysis plan

### General principles

All analyses will be based on the intention-to-treat (ITT) principle. CONSORT guidelines, for feasibility studies, will be followed [5, 6].

### Interim analyses

No formal interim analysis is planned. Recruitment data will be available to the TAPS2 Trial Steering Committee (TSC) as the study continues. The full length of the study is designed to be sufficient to determine whether the main study is feasible.

### Main analysis

Our primary outcome (overall recruitment rate as a percentage of eligible women) will be estimated within 10% of the true value or better using the Clopper-Pearson exact Binomial method for a 95% confidence interval. The number of women screened, eligible, consented, randomised and withdrawn from the study will be reported by site, and overall numbers summarised as a CONSORT flow diagram. Reasons for exclusion and for withdrawal will be summarised. Adherence to the intervention will be assessed by the number of SPEBTs received by women randomised to the intervention arm and will be presented in the CONSORT flow diagram.

Descriptive statistics including 95% confidence intervals will be presented for all baseline data and clinical outcomes, with a focus on estimates of standard deviation necessary to perform sample size calculations for a future trial.

Although the study is expected to be underpowered for clinical outcomes, the differences in the clinical outcomes will be presented as an initial assessment of the efficacy and safety of this treatment, and for inclusion in any future meta-analysis. Clinical outcomes will be analysed using a complete case analysis approach. No subgroup analyses are planned.

We plan to conduct a single analysis at the end of data collection. A set of dummy tables for all feasibility and clinical outcomes are provided in Additional file 1.

### Methods for handling missing data

Every effort will be made to obtain complete data where possible. Levels of missingness will be reported for all outcome variables, as a percentage with 95% CI, and used as estimates of the likely level of missingness in the main study. No formal adjustment will be made for missingness in the analysis of outcome data, as the numbers of events are likely to be too small for this to be feasible.

### Software

A study-specific internet-based secure data management system (MedSciNet™) is the repository for all trial data. Baseline and follow-up data will be entered on to the MedSciNet database contemporaneously, with the exception of pregnancy outcome follow-up which will be updated postnatally. The study statistician will extract data from the database as required. Statistical analyses will be performed using Stata Version 16 or later (StataCorp, College Station, TX, USA).

### Statistical reporting conventions

Percentages will be rounded and presented to the nearest whole number. Continuous measures (such as averages and standard deviations) will be reported to two or (where appropriate) three significant figures. Arithmetic means (and SD) will be presented for continuous variables which are approximately normally distributed; medians (quartiles) otherwise. For counts of rare events (experienced by most participants), frequencies will be tabulated, and results compared by ordered logistic regression, leading to risk ratios.

Comparisons between treatment groups will be presented with 95% confidence intervals, and standard errors. Conventional significance will be taken at *P* < 0.05. However, the main emphasis of the research will be on producing useful estimates for planning a future study.

## Qualitative study

### Aim

The aim of the nested qualitative study is to capture the views and experiences of trial participants, decliners and clinical staff involved in the study, to inform the design of a definitive trial with a particular focus on optimising recruitment strategies.

### Summary

Semi-structured interviews will be conducted with three groups: trial participants; women who were eligible but unwilling to participate in the trial; and clinical staff involved in trial recruitment and intervention delivery. Interviews will explore the acceptability of intervention (participants); views on trial procedures and study conduct including outcome measures (participants and clinicians) and acceptability of randomisation (participants); views on barriers and facilitator to participation in the trial (participants, those unwilling to participate and clinicians); reasons for attrition (participants and clinicians); and identify strategies to optimise recruitment and retention for the definitive trial (participants, clinicians and those unwilling to participate). We will follow recent guidance regarding the effective use of qualitative research in feasibility studies for RCTs [10].

### Methods and recruitment

#### Interviews with study participants

Semi-structured interviews will be conducted with between 15 and 25 women recruited from the trial population. Written informed consent for interviews will be obtained at the same time as participants are consented into the trial. Interviews will be conducted around 6–8 weeks post-partum, will take place either face-to-face or by telephone, and will last approximately 30–45-min duration. Using a maximum diversity sampling approach, we will aim to recruit women who: completed the study and those who dropped out; those from different recruitment sites; those for whom this is their first birth versus those who had previous pregnancies; those with different genotypes, which may affect treatment experience.

#### Interviews with women who decline to participate in the trial 

We aim to interview between 5 and 15 women who are eligible to join the feasibility trial but decline to participate (“decliners”). Written consent will be obtained by the recruiting staff member, and participants will subsequently be contacted by a qualitative researcher to arrange a telephone interview lasting approximately 15–20-min duration.

#### Interviews with clinical staff

We will aim to interview two to three staff considered ‘key informants’ in each study centre (e.g. research nurses/midwives, sickle nurse specialists, sickle haematologists and sickle obstetricians) to inform the definitive trial. Interviews will be conducted once the site recruitment phase has been completed (or if not possible, towards the end of the recruitment phase) to enable participants to have had adequate experience to reflect on the trial. Telephone interviews will be conducted; the duration of interviews will be approximately 30–45 min.

Topic guides will be devised for the three sets of qualitative interviews. Consistent with inductive qualitative research, relevant topics that arise during interviews will be added to the topic guide.

### Analysis

All interviews will be digitally recorded and transcribed verbatim, and anonymised transcripts uploaded onto NVivo for Windows (Release 1.3) for data management and coding. Illustrative quotes will be reported to support themes and subthemes for each data set; pseudonyms will be used to ensure anonymity.

Interviews with trial participants will be analysed using reflexive thematic analysis [11], using the constant comparison approach to compare data within and across individual interviews to identify themes and subthemes. We will undertake subgroup analysis to identify any differences in findings across study sites, those who completed the study versus dropouts, previous pregnancy history, and different SCD genotypes. Standard methods will be employed to ensure rigour (e.g. audit trail, reflexive diaries, double coding, deviant case analysis).

Interviews with staff will be analysed as described for the trial participants, including sub-group analysis to identify differences in findings across study sites.

Data from interviews with women who declined to participate will be extracted from repeated listening of the audio files and incorporated into summary tables to report reasons for declining, suggestions to improve recruitment, and views on the preference of study design for the definitive trial. Additionally, differences in responses will be explored to determine if pregnancy history or previous history of transfusion, or concerns about the COVID-19 pandemic affects these participants’ decision to participate in this trial.

## Conclusion

This statistical and qualitative analysis plan describes how the quantitative and qualitative data from this feasibility trial will be presented and analysed. This analysis plan was submitted to the journal prior to analysis to aid transparency, reduce reporting bias, and increase the validity of the study results.

### Current trial status and further information

Recruitment to TAPS2 ended on October 31, 2022. Final follow-up data collection is due to take place in May 2023. The current version of the TAPS2 protocol is v1.3 13.08.2021. This analysis plan was formally approved at the TAPS2 Trial Steering Committee meeting on 12th January 2023. The Trial Master File, standard operating procedures and the study handbooks are stored securely at the TAPS2 coordinating centre located at Guy’s and St Thomas’ NHS Foundation Trust.

## Supplementary Information

**Additional file 1: Table S1.** Eligibility, recruitment to RCT, and trial completion. **Table S2.** Description of women at trial entry. **Table S3.** Maternal primary and secondary outcomes (post randomisation). **Table S4.** Neonatal outcomes. **Table S5.** Safety outcomes. **Table S6.** Follow-up assessment at 6 weeks post-partum.

## Data Availability

Not applicable.

## Abbreviations

BSH: British Society for Haematology
PIS: Patient Information Sheet
PPI: Patient and Public Involvement
RCOG: Royal College of Obstetricians and Gynaecologists
RCT: Randomised controlled trial
SCD: Sickle cell disease
SPEBT: Serial prophylactic exchange blood transfusion
TSC: Trial Steering Committee
