# Supplementary material for: Serial prophylactic exchange blood transfusion in pregnant women with sickle cell disease (TAPS-2): statistical and qualitative analysis plan for a randomised controlled feasibility trial
Source: Trials. 2023 Mar 24;24:225. doi: 10.1186/s13063-023-07235-x (PMC10037353; doi:10.1186/s13063-023-07235-x)
Supplement: Supplementary file 1 — Additional file 1: Table S1. Eligibility, recruitment to RCT, and trial completion. Table S2. Description of women at trial entry. Table S3. Maternal primary and secondary outcomes (post randomisation). Table S4. Neonatal outcomes. Table S5. Safety outcomes. Table S6. Follow-up assessment at 6 weeks post-partum. [file 13063_2023_7235_MOESM1_ESM.docx]

# ADDITIONAL FILE 1

| **Centre number** | **1** | **2** | **3** | **… 7** | **All centres** |
| --- | --- | --- | --- | --- | --- |
| **Length of recruitment (months)** | M | M | M | M | Total M |
| **Total number**  **of pregnancies** | N | N | N | N | Total N |
| **% eligible** | n/N (%)  (95% CI) | n/N (%)  (95% CI) | n/N (%)  (95% CI) | n/N (%)  (95% CI) | n/N (%)  (95% CI) |
| **% of eligible recruited** | n/N (%)  (95% CI) | n/N (%)  (95% CI) | n/N (%)  (95% CI) | n/N (%)  (95% CI) | n/N (%)  (95% CI) |
| **Recruitment per centre per month** | Rate | Rate | Rate | Rate | Mean rate (SD) |
| **% of recruited with complete primary data** | n/N (%)  (95% CI) | n/N (%)  (95% CI) | n/N (%)  (95% CI) | n/N (%)  (95% CI) | n/N (%)  (95% CI) |

Table 1: Eligibility, recruitment to RCT, and trial completion

Table 2: Description of women at trial entry

|  | **Usual care**  **(N=)** | **Intervention**  **(N=)** | **All women**  **(N=)** |
| --- | --- | --- | --- |
| **Age (years)** | mean (SD) | mean (SD) | mean (SD) |
| **Parity**  **Primiparous** | n (%) | n (%) | n (%) |
| **BMI** | mean (SD) | mean (SD) | mean (SD) |
| **Gestation at recruitment (weeks)** | mean (SD) | mean (SD) | mean (SD) |
| **Clinical disease**  **HbSS**  **HbSC**  **Other** | n (%)  n (%)  n (%) | n (%)  n (%)  n (%) | n (%)  n (%)  n (%) |

Table 3: Maternal primary and secondary outcomes (post randomisation)

|  | **Usual care**  **(N=)** | **Intervention**  **(N=)** | **Comparison** |
| --- | --- | --- | --- |
| **HbS% (pre-transfusion), where recorded** | n/a | N mean (SD) | n/a |
| **HbS% (post-transfusion)** | n/a | N mean (SD) | n/a |
| **Hospital admission**  **(any)** | n (%) | n (%) | Risk Ratio (95% CI) |
| **Hospital admission  (SCD-related)** | n (%) | n (%) | Risk Ratio (95% CI) |
| **Hospital admission  (non SCD-related)** | n (%) | n (%) | Risk Ratio (95% CI) |
| **Length of stay**  **(if admitted)** | Median (quartiles) | Median (quartiles) | Difference (95% CI) |
| **Sickle cell crisis (any)**  **1 or more** | n (%) | n (%) | Risk Ratio (95% CI) |
| **Severe or extremely severe sickle cell crisis***  **1 or more** | n (%) | n (%) | Risk Ratio (95% CI) |
| **Acute chest syndrome** | n (%) | n (%) | Risk Ratio (95% CI) |
| **Preeclampsia** | n (%) | n (%) | Risk Ratio (95% CI) |
| **VTE/PE** | n (%) | n (%) | Risk Ratio (95% CI) |
| **Induction of labour** | n (%) | n (%) | Risk Ratio (95% CI) |
| **Mode of birth**  **Emergency caesarean**  **Elective caesarean** | n (%)  n (%) | n (%)  n (%) | Risk Ratio (95% CI)  Risk Ratio (95% CI) |
| **Ad-hoc top-up transfusion**  **1 or more** | n (%) | n (%) | Risk Ratio (95% CI) |
| **Ad-hoc exchange transfusion**  **1 or more** | n (%) | n/a | n/a |

** The definition of painful crisis was divided into mild, moderate, severe and extremely severe crisis. Mild crises may or may not have required pain medication but did not prevent normal activity, moderate crises required medication and caused significant changes in daily activities, severe crises required attendance at hospital and extremely severe crises required hospital admission.*

Table 4: Neonatal outcomes

|  | **Usual care**  **(N=)** | **Intervention**  **(N=)** | **Comparison** |
| --- | --- | --- | --- |
| **Birthweight (gm)** | mean (SD) | mean (SD) | Difference (95% CI) |
| **Premature delivery <37 weeks** | n (%) | n (%) | Risk Ratio (95% CI) |
| **SGA (<10^th^ centile)** | n (%) | n (%) | Risk Ratio (95% CI) |
| **NICU/CC admission** | n (%) | n (%) | Risk Ratio (95% CI) |
| **Stillbirth** | n (%) | n (%) | Risk Ratio (95% CI) |
| **Breast feeding at discharge** | n (%) | n (%) | Risk Ratio (95% CI) |
| **Apgar < 7 at 5 minutes** | n (%) | n (%) | Risk Ratio (95% CI) |

Table 5: Safety outcomes

|  | **Usual care**  **(N=)** | **Intervention**  **(N=)** | **Comparison** |
| --- | --- | --- | --- |
| **Any transfusion reactions** | n (%) | n (%) | Risk Ratio (95% CI) |
| **Number of reactions** | mean (SD) | mean (SD) | Difference (95% CI) |
| **Alloimmunisation** | n (%) | n (%) | Risk Ratio (95% CI) |
| **Delayed haemolytic transfusion reaction** | n (%) | n (%) | Risk Ratio (95% CI) |

Table 6: Follow-up assessment at 6 weeks post-partum

|  | **Usual care**  **(N=)** | **Intervention**  **(N=)** | **Comparison** |
| --- | --- | --- | --- |
| **Postpartum symptoms**  **Pain**  **Sickle cell crisis**  **Chest infection**  **Pulmonary embolism**  **Other *** | n (%)  n (%)  n (%)  n (%)  n (%) | n (%)  n (%)  n (%)  n (%)  n (%) | Risk Ratio (95% CI)  Risk Ratio (95% CI)  Risk Ratio (95% CI)  Risk Ratio (95% CI)  Risk Ratio (95% CI) |
| **Hospital admissions**  **unrelated to SCD (nights)** | Median (quartiles) | Median (quartiles) | Difference (95% CI) |
| **Hospital admissions**  **related to SCD (nights)** | Median (quartiles) | Median (quartiles) | Difference (95% CI) |
| **Postnatal transfusions** | n (%) | n (%) | Risk Ratio |
| **Number of transfusions**  **0**  **1**  **2** | n (%)  n (%)  n (%) | n (%)  n (%)  n (%) | Risk Ratio (95% CI) |
| **Day unit attendance**  **0**  **1**  **2** | n (%)  n (%)  n (%) | n (%)  n (%)  n (%) | Risk Ratio (95% CI) |
